# Connaissances, attitudes et pratiques des étudiants en médecine en matière de sexualité

**DOI:** 10.11604/pamj.2020.35.141.12910

**Published:** 2020-04-29

**Authors:** Imen Baati, Sahar Ellouze, Jihen Jedidi, Rim Sellami, Dorsaf Trigui, Jamel Damak, Ines Feki, Jawaher Masmoudi

**Affiliations:** 1 Service de psychiatrie «A», CHU Hédi Chaker Sfax, Université de Sfax, Tunisie; 2 Service de médecine communautaire et d’épidémiologie, CHU Hédi Chaker Sfax, Université de Sfax, Tunisie

**Keywords:** Sexualité, étudiant, connaissances, attitudes, comportement sexuel, Sexuality, student, knowledge, attitudes, sexual behavior

## Abstract

La sexualité est un aspect central de la personne humaine qui, devant des problèmes sexuels, va fréquemment se tourner vers une source qu'elle considère compétente et bien informée, son médecin. Les objectifs de notre étude étaient d’évaluer les connaissances, les attitudes et les pratiques des étudiants en médecine en termes de sexualité et d’identifier les principaux déterminants du manque de connaissances basiques à ce sujet. Notre étude était descriptive et analytique, menée auprès des étudiants à la faculté de médecine de Sfax (Tunisie). Le questionnaire, rempli individuellement et de manière anonyme par chaque étudiant, recueillait les données sociodémographiques, les connaissances en matière de sexualité ainsi que leurs sources, les attitudes adoptées par les étudiants vis-à-vis de la sexualité et les pratiques sexuelles. La note totale moyenne relative aux questions de sexologie était de 11,05/20. Les facteurs corrélés à un score moyen élevé aux questions de sexologie étaient le sexe masculin (p=0,003), le statut de marié (p=0,012), le niveau socio-économique élevé (p=0,02), les livres comme source d’informations (p=0,041) et la présence de pratiques sexuelles (p<0,001). Ces pratiques étaient toutes significativement plus fréquentes chez les étudiants de sexe masculin (p<0,001). Des lacunes dans les connaissances en matière de sexualité existent chez les étudiants en médecine, du moins dans certains de ses aspects. Un programme d'enseignement complet et uniforme sur la sexualité humaine, surtout dans ses aspects physiologiques, peut sensiblement améliorer la capacité des futurs médecins à fournir des soins optimaux à leurs patients.

## Abstract (en)

Sexuality is a central aspect of being human throughout life. People with sexual problems commonly turn to their doctor, because he is regarded as competent and well informed. The purpose of our study was to assess the knowledge, attitudes and practice of medical students concerning sexual matters and to identify the main determinants of the lack of basic knowledge on this subject. We conducted a descriptive and analytical study of students in the Faculty of Medicine of Sfax (Tunisia). The questionnaire, completed individually and anonymously by each student, collected sociodemographic data, knowledge about sexuality as well as sources, student's attitude towards sexuality and sexual practices. The overall average score of sexology related questions was 11,05/20. Factors correlated with a medium high score of sexology related questions were: male sex (p=0.003), married status (p=0.012), high socioeconomic status (p=0.02), books as a source of the information (p=0.041) and sexual practices (p<0.001). Sexual practices were significantly more frequent among male students (p<0.001). There are gaps in knowledge about sexuality among medical students, at least in some of its aspects. A complete and uniform educational program about human sexuality, especially in its physiological aspects, can significantly improve the ability of future physicians to provide optimal patient care.

## Introduction

La sexualité est un aspect central de la personne humaine tout au long de sa vie [1]. Selon l'Organisation mondiale de la santé (OMS) [2], la santé sexuelle fait partie intégrante de la santé, du bien-être et de la qualité de vie dans leur ensemble. Les personnes demandant des conseils ou des soins pour leurs problèmes sexuels vont fréquemment se tourner vers une source qu'elles considèrent compétente, bien informée et digne de respect, leur médecin [3]. Ainsi, un futur médecin devrait acquérir des connaissances basiques en sexologie qui lui permettraient d’être plus à l’aise dans les consultations et d’évaluer les pratiques sexuelles des patients sans jugement moral [4]. Toutefois, plusieurs études [5,6] ont montré que, pendant l'enseignement universitaire ou postuniversitaire en médecine, la formation est variable, non standardisée, ou encore inadéquate en matière de sexualité humaine et de ses troubles. Les objectifs de notre étude étaient d’évaluer les connaissances, les attitudes et les pratiques des étudiants en médecine en termes de sexualité et d’identifier les principaux déterminants du manque de connaissances basiques concernant la sexualité.

## Méthodes

**Population:** sur 220 étudiants en médecine sollicités pour l’étude, 100 (soit 45,5%) ont refusé d’y participer. L’étude a donc porté sur 120 étudiants de différents niveaux (de la première à la cinquième année ainsi que des internes en première et en deuxième année).

**Questionnaire:** un questionnaire auto-administré a été rédigé en français et soumis aux étudiants. Les informations recueillies étaient réparties en quatre rubriques. La première concernait des données sociodémographiques. La deuxième s’intéressait aux connaissances en matière de sexualité, ainsi que leurs sources. Les items évaluant les connaissances étaient inspirés d’un cours de sexologie enseigné dans le cadre du mastère professionnel en sexologie, à la faculté de médecine de Sfax, au cours de l’année universitaire 2010-2011. Ces items, au nombre de 20, étaient sous forme de questions à réponses binaires (vrai ou faux). Leur contenu correspondait soit à des données scientifiquement valides, soit à des erreurs cognitives répandues dans le milieu socioculturel de l’étude. Chaque réponse correcte étant cotée 1, l’étudiant avait une note allant de 0 à 20. Les thèmes évalués étaient ceux concernant la masturbation (3 items: 1 à 3), la sexualité à l´adolescence (2 items: 4 et 5), l´acte sexuel (9 items: 6 à 14), la sexualité et la grossesse (4 items: 15 à 18) et la sexualité des aînés (2 items: 19 et 20). La troisième rubrique était consacrée aux attitudes adoptées par les étudiants vis-à-vis de la sexualité: l´abord de la sexualité dans leurs discussions, l´importance accordée à la sexualité, le modèle sociétal préféré et les raisons d´une activité sexuelle réduite. Finalement, la quatrième rubrique décrivait les pratiques sexuelles: la masturbation, les rapports sexuels (superficiels ou pénétrants).

**Procédure:** il s’agissait d’une évaluation transversale, descriptive et analytique, qui s’est déroulée à la faculté de médecine de Sfax, au cours du mois de décembre 2012. Chaque participant recruté a obtenu des informations sur le contenu de l’évaluation, sur la confidentialité du recueil et du traitement des données, et sur la liberté de sa participation à l'étude.

**Méthodes statistiques:** l’analyse statistique a été réalisée avec le logiciel SPSS (Statistical Package for Sciences) dans sa 20^ème^ version. L'étude des associations entre les variables a été faite par les tests d´hypothèses. La comparaison des proportions a été réalisée par le test « *Chi2* » de Pearson ou par le test exact de Fisher. Le test de Student a été utilisé pour la comparaison de deux moyennes de deux échantillons indépendants. Le seuil de significativité retenu était de 5%.

## Résultats

**Caractéristiques sociodémographiques:** sur les 120 étudiants interrogés, 115 (soit 95,8%) étaient célibataires et 75 (soit 62,5%) étaient des filles. L´âge des participants variait de 18 à 30 ans avec une moyenne de 22,33 ans. Ils étaient des étudiants du premier cycle (1^ère^ et 2^ème^ année) dans 26,7% des cas (N=32), et du deuxième cycle (3^ème^, 4^ème^, 5^ème^ année et internes) dans 73,3% des cas (N=88). Les caractéristiques sociodémographiques des participants étaient résumées dans le Tableau 1.

**Tableau 1:** Caractéristiques sociodémographiques des participants

		Effectif	Pourcentage
**Sexe**			
	Masculin	45	37,5
	Féminin	75	62,5
**Statut matrimonial**			
	Célibataire	115	95,8
	Marié	5	4,2
**Origine géographique**			
	Urbaine	88	73,3
	Semi-urbaine	25	20,8
	Rurale	7	5,9
**Mode d’habitation**			
	Avec les parents	91	75,8
	En colocation	16	13,3
	Seul	8	6,7
	Avec conjoint	5	4,2
**Niveau socio-économique**			
	Moyen	100	83,3
	Elevé	20	16,7
**Niveau d’études**			
	1ère année	11	9,2
	2ème année	21	17,5
	3ème année	16	13,3
	4ème année	20	16,7
	5ème année	21	17,5
	Interne 1ère année	16	13,3
	Interne 2ème année	15	12,5

### Connaissances sur la sexualité

**Sources d´informations:** les sources d'informations sur la sexualité chez nos étudiants étaient essentiellement les amis (69,2%; N=83), Internet (58,3%; N=70) et la télévision (47,5%; N=57). Les autres sources étaient les enseignants (35%; N=42), les livres (24,2%; N=29), les parents (14,2%; N=17) et la fratrie (10,8%; N=13).

**Connaissances:** la note globale attribuée au questionnaire de sexologie variait de 0 à 19 sur 20, avec une moyenne de 11,05 (±4,24). Les facteurs corrélés à une note moyenne élevée étaient le sexe masculin (12,53 vs 10,14; p = 0,003), le statut de marié (13,8 vs 10,93 ; p = 0,012), le niveau socioéconomique élevé (13,11 vs 10,64; p = 0,02) et les livres comme source d'informations (12,54 vs 10,61 ; p = 0,041). Plus de la moitié des participants ont fourni des réponses incorrectes pour huit items (Tableau 2). Le taux moyen de réponses correctes au questionnaire était de 52%.

**Tableau 2:** Réponses des participants au questionnaire de sexologie

Items	Nombre de réponses (%)	
	correctes	incorrectes
La masturbation est un phénomène pathologique.	65(54,2)	55(45,8)
La masturbation est nuisible pour la santé.	44(36,7)	76(63,3)
La masturbation chez la fille peut lui faire perdre sa virginité.	44(36,7)	76(63,3)
L’éducation sexuelle des enfants les incite à la débauche.	75(62,5)	45(37,5)
Tout adolescent présentant une tendance homosexuelle sera un homosexuel à l’âge adulte.	66(55)	54(45)
L’attitude active de la femme lors d’un rapport sexuel est un signe de relâchement moral.	49(40,8)	71(59,2)
L’acte sexuel n’est satisfaisant pour les deux partenaires que si l’orgasme est simultané.	28(23,3)	92(76,7)
Tout comme l’homme, la femme a une période réfractaire.	47(39,2)	73(60,8)
La femme doit simuler l’orgasme pour ne pas heurter la fierté de l’homme.	57(47,5)	63(52,5)
La défloration s’accompagne obligatoirement d’une douleur intense et d’un écoulement sanguin très abondant.	88(73,3)	32(26,7)
L’homme doit obligatoirement déflorer son épouse lors de la nuit de noces.	84(70)	36(30)
Le plaisir de la femme est proportionnel à la taille et à la grosseur du pénis du partenaire.	81(67,5)	39(32,5)
La performance sexuelle est proportionnelle aux dimensions du pénis.	77(64,2)	43(35,8)
Plus l’homme est viril, plus son éjaculation est rapide.	76(63,3)	44(36,7)
Pendant toute la période de la grossesse, il y a une baisse du désir sexuel.	60(50)	60(50)
Lors des rapports sexuels, le fœtus peut être blesse ou peut souffrir.	71(59,2)	49 (40,8)
Les rapports sexuels causent des fausses couches et des accouchements prématurés.	59(49,2)	61(50,8)
Les rapports sexuels ne peuvent être repris que 40 jours après l’accouchement.	24(20)	96(80)
La ménopause est incompatible avec une sexualité satisfaisante chez la femme.	61(50,8)	59(49,2)
La sexualité de l’homme (ou de la femme) doit s’arrêter quand il (elle) devient âgé(e).	100(83,3)	20(16,7)

**Attitudes:** la discussion à propos de sexualité a déjà été abordée par 93,3% des participants (N=112). Cette discussion était appréciée par 45,8% d´entre eux (N=55). Elle était considérée comme « gênante » dans 12,5% des cas (N= 15). Les participants accordaient une importance « particulière » à la sexualité dans 30,8% des cas (N=37); cette importance était « normale » dans 59,2% (N= 71), « minime » dans 6,7% des cas (N= 8) et « nulle » dans 3,3% des cas (N= 4). L'intérêt pour la sexualité s´est manifesté à un âge moyen de 15,47 ans (ET=2,94 ans). Plus de la moitié des enquêtés (59,2%; N=71) préféraient une société qui interdisait les relations sexuelles hors mariage. Ils avaient une sexualité réduite « par principe » dans 28,3% des cas (N=34).

**Pratiques sexuelles:** la masturbation était la pratique sexuelle la plus répandue chez nos étudiants (N=53; 44,2%). Parmi les étudiants célibataires (N=115), 26,1% (N=30; 22 garçons et 8 filles) ont eu des rapports sexuels superficiels et 12,2% (N=14; tous des garçons) ont eu des rapports sexuels pénétrants. L´âge moyen au premier rapport pénétrant était de 16,86 ans (ET = 2,93 ans). Toutes les pratiques sexuelles étaient significativement corrélées au sexe masculin (p < 0,001 pour chaque pratique).

**Relation entre connaissances, attitudes et pratiques sexuelles:** les étudiants ayant pratiqué la masturbation ou ayant eu des rapports sexuels superficiels ou pénétrants avaient des notes globales significativement plus élevées. La même constatation était faite avec la note spécifique des items concernant la « masturbation » (Tableau 3). Les étudiants qui préféraient un modèle sociétal interdisant les rapports sexuels hors du cadre du mariage avaient des pratiques sexuelles significativement moins fréquentes (p < 0,001 pour la masturbation, les rapports sexuels superficiels et les rapports sexuels pénétrants). La discussion à propos de la sexualité était plus appréciée par les participants ayant pratiqué une masturbation (p = 0,028) ou un rapport sexuel superficiel (p = 0,045).

**Tableau 3:** Corrélation entre connaissances en sexologie et pratiques sexuelles

		Pratiques					
C O N N A I S S A N C E S	**Notes**	**Masturbation**		**Rapport sexuel superficiel**		**Rapport sexuel pénétrant**	
		Oui	Non	Oui	Non	Oui	Non
	Globale (/20)	12,75	9,42	13,55	10,11	13,44	10,66
	p	<0,001		<0,001		0,015	
	« Masturbation » (/3)	1,88	1,09	2,03	1,24	2,12	1,36
	p	0,001		0,001		0,001	
	« Sexualité de l’adolescent » (/2)	1,47	1,25	1,41	1,35	1,6	1,33
	p	0,148		0,71		0,1	
	« Acte sexuel » (/9)	6,04	5,48	6,5	5,51	6,23	5,78
	p	0,26		0,043		0,47	
	« Sexualité et grossesse » (/4)	2,1	2,21	2,2	2,08	2,06	2,13
	p	0,65		0,16		0,83	
	«Sexualité des ainés » (/2)	1,62	1,35	1,58	1,47	1,56	1,49
	p	0,027		0,4		0,68	

## Discussion

Notre étude offre un aperçu global des connaissances des étudiants en médecine en matière de sexualité, et permet de souligner certaines particularités de leurs attitudes et pratiques sexuelles.

**Taux de participation:** le taux de réponse des étudiants sollicités pour l´étude (45,4%) était légèrement inférieur à celui trouvé par Turner *et al*. [7] dans leur étude portant sur les connaissances des étudiants en médecine en Allemagne à propos de la sexualité humaine (52,9%). Notre résultat était, par ailleurs, comparable à celui rapporté par Masmoudi-Soussi *et al*. [8] dans leur étude portant sur la vie sexuelle de 352 étudiants tunisiens de la région de Sfax (44%). En effet, le tabou entourant le thème de la sexualité serait bien universel, mais il est particulièrement prépondérant dans les sociétés musulmanes, où pudeur et chasteté conservent toujours une place importante [9].

**Connaissances et sources d´informations sur la sexualité:** le taux moyen de réponses correctes au questionnaire était de 52%. Ce résultat se rapproche de celui trouvé dans l'étude menée par Turner *et al*. en 2012 [7], avec 50,3% de réponses correctes. Des études antérieures [10,11] pointent dans la même direction, avec des étudiants en médecine donnant entre 60% et 80% de réponses correctes dans les questionnaires de connaissance sur la sexualité. Il serait difficile de comparer ces taux de réponses puisque le contenu des questionnaires était très variable d'une étude à une autre. Dans notre travail, les items ayant reçu le moins de réponses correctes étaient ceux relatifs à la masturbation, à l´acte sexuel et à la sexualité au cours de la grossesse. En effet, 45,8% de nos étudiants considéraient la masturbation comme un phénomène pathologique et 63,3% d´entre eux jugeaient cette conduite comme étant nuisible pour la santé et pouvant faire perdre la virginité. Ces données, qui s´accordent avec celles relevées chez les étudiants en médecine en Malaisie [12], seraient attendues dans notre contexte arabo-musulman puisque le jeune qui parvient à satisfaire ses besoins sexuels par la masturbation croit, très souvent, qu´il a tort sur le plan physique, moral, et surtout religieux, ce qui accroît son sentiment de honte et de culpabilité [8].

Concernant les items relatifs à l´acte sexuel, les questions se rapportant au rôle de la femme lors des rapports sexuels ont reçu le moins de réponses correctes. En effet, l´attitude active de la femme lors d´un rapport sexuel était jugée comme un signe de relâchement moral par 59,2% des étudiants. Dans l´étude de Ben Thabet *et al*. [13] portant sur la sexualité de la femme tunisienne, 43,6% des participants considéraient que la femme devrait rester passive lors des rapports sexuels. Cette idée que les hommes doivent être actifs et les femmes passives et soumises découle généralement des valeurs inculquées par les ascendants et les mythes [14]. Exhiber la passion est honteux pour une fille [15]. Par ailleurs, une femme mariée qui exprime son désir sexuel peut être stigmatisée et considérée comme non respectable dans certains pays arabes [9]. Pour les items relatifs à la sexualité et la grossesse, la question qui se rapporte à la reprise de l´activité sexuelle au post-partum a eu le taux le plus faible de réponses correctes (20%). Selon la majorité des enquêtés, les rapports sexuels ne peuvent être repris que 40 jours après l´accouchement. Une telle réflexion est en rapport avec les pesanteurs socioculturelles et religieuses qui considèrent que les femmes sont impures pendant toute la période des pertes sanguines du post-partum. Or, scientifiquement parlant, la durée de cette période est très variable d´une femme à l´autre. Selon la religion islamique, une femme peut reprendre son activité sexuelle au post-partum dès la disparition totale du saignement. Le délai minimal de 40 jours, souvent rapporté dans notre contexte, n´est que le fruit d´un héritage socioculturel. D’après de Pierrepont *et al*. [16], la durée de l´abstinence sexuelle postnatale est très variable, de l´ordre de quelques jours à quelques semaines et peut atteindre plusieurs années dans certaines cultures.

Les mauvaises notes obtenues dans la présente étude nous amènent inéluctablement à réfléchir sur les sources d´informations de nos étudiants concernant la sexualité humaine. Nous rappelons que 65% de nos étudiants ne considéraient pas les enseignants comme source d´informations sur la sexualité, encore moins les livres (75,8%). Des résultats similaires ont été signalés dans l´étude de Turner *et al*. [7]. C´est pourquoi, nous suggérons que les réponses données par nos participants n´étaient pas basées sur des données scientifiques acquises lors des cours magistraux, ni sur une source scientifique fiable. Elles étaient plutôt des réponses inspirées des idées reçues transmises par les amis et les médias (Internet et télévision), principales sources d´informations dans notre étude, et dans d´autres études tunisiennes [8] et internationales [17-19]. Quoique peu nombreux, les étudiants qui consultaient des livres pour améliorer leurs connaissances sur la sexualité (24,2%) avaient une note globale meilleure (p= 0,041). Ceci dit, les livres, notamment scientifiques, sont une source fiable pour les étudiants en médecine. Ce n´est pas le cas d'Internet, la seconde source de connaissances dans notre étude. D´ailleurs, Zdanowicz et Reynert [4] ont rapporté que l´usage d´Internet à des fins sexuelles n´avait pas d´influence sur le score au questionnaire de sexologie et ont conclu que l´idée populaire qu´Internet transforme les jeunes en « experts » du sexe était sans fondement. Quant aux parents, ils ont été quasi-exclus en tant que source d'informations dans notre étude (14,2%). Ceci a été également noté dans l´enquête menée par Ayedi [20], portant sur la sexualité de 100 lycéens de la région de Sfax, où 94,5% des lycéens enquêtés écartaient les parents en tant que source d´informations au sujet de la sexualité. Ce phénomène paraît courant dans notre contexte socioculturel puisque la sexualité est encore considérée comme un sujet tabou, surtout dans le dialogue entre générations [8]. En plus de ces sources d´informations déjà rapportées par nos étudiants, il semblerait que leur propre expérience sexuelle leur a permis d'améliorer leurs connaissances sur la sexualité, comme en témoigne la corrélation entre la note globale et toutes les pratiques sexuelles. En se basant sur cette hypothèse, nous pouvons également expliquer la relation entre la note globale et le sexe masculin et le statut de marié. Ces deux facteurs étaient corrélés à une note globale élevée, puisqu´il s´agit de personnes qui avaient eu plus de rapports sexuels.

**Attitudes en matière de sexualité:** dans notre étude, la quasi-totalité des étudiants (93,3%) ont déjà abordé le sujet de la sexualité dans leurs discussions. La discussion à propos de la sexualité a été jugée gênante dans 12,5% des cas. Nos résultats s´accordent avec ceux trouvés par Aggarwal *et al*. [17], dans leur enquête menée auprès des étudiants en médecine en Inde, révélant que 88,5% des participants avaient déjà discuté de sexualité, et que 20% d'entre eux éprouvaient des difficultés à parler de sexualité avec le sexe opposé. Ces étudiants en médecine, qui n´arrivent pas à surmonter leur gêne en parlant de sexualité, seraient probablement influencés par le tabou qui entoure ce sujet dans leurs sociétés. Concernant la relation sexuelle avant le mariage, les opinions de nos étudiants vont dans le même sens que ceux des enquêtés de l´étude de Masmoudi-Soussi *et al*. [8], où cette relation a été jugée inacceptable par 58% des participants, surtout de sexe féminin, pour des considérations religieuses et éthiques au premier plan. D'ailleurs, le modèle sociétal interdisant les rapports sexuels hors du cadre du mariage était le modèle préféré de 59,2% de nos étudiants. Les étudiants en médecine en Malaisie seraient du même avis puisque 80,8% parmi eux désapprouvaient les relations sexuelles avant le mariage [12]. Cette attitude conservatrice est régie par la religion officielle, l´islam, qui comme toutes les religions monothéistes, n´autorise les relations sexuelles que dans le cadre strict du mariage.

**Pratiques sexuelles:** dans notre série, les pratiques de la masturbation et de rapport sexuel superficiel ou pénétrant se rapprochent des résultats issus d´autres études réalisées également dans la région de Sfax [8,21]. Ainsi, les comportements sexuels des étudiants en médecine dans cette région n´ont pas changé au fil des années, entre 1999 [21] et 2012; ils ne diffèrent pas non plus des autres étudiants des autres facultés [8]. Toutefois, ces résultats sont largement à l´écart de ceux retrouvés dans les études occidentales. Alors que la pratique de la masturbation a été reconnue par 44,2% des participants à notre étude, Breyer *et al*. [22], dans leur étude réalisée auprès des étudiants en médecine en Amérique du Nord, ont rapporté des taux supérieurs à 80%. Un résultat similaire a été trouvé chez des étudiants en médecine de sexe masculin en Turquie [23]. Concernant la pratique de rapports sexuels, 26,1% de nos étudiants célibataires ont reconnu avoir eu des rapports sexuels non pénétrants, rejoignant ainsi leurs homologues en Inde (23,3%) [17]. Par ailleurs, 12,2% de nos participants célibataires ont déclaré avoir eu des rapports sexuels pénétrants. Un tel pourcentage se rapproche de celui trouvé chez les étudiants en 3ème année médecine au Sud de la Chine dont 19,6% étaient sexuellement actifs [24].

Cependant, une étude récente menée par Bulot *et al*. [25] et portant sur la pornographie et les comportements sexuels en milieu universitaire, 88% des étudiants ont eu des relations sexuelles, en proportion quasi-égale chez les hommes et les femmes. Cette similitude des pratiques sexuelles entre les deux sexes n´a pas été retrouvée dans les études turques [26], ni tunisiennes [8] y compris la nôtre, où toutes les pratiques sexuelles se sont avérées significativement plus élevées chez les étudiants de sexe masculin. Ce résultat serait lié aux normes sociales qui prévalent dans les pays arabes et/ou musulmans comme la Tunisie, où la sexualité féminine reste entravée par les interdits de la religion et de la culture. S´ajoute à cela la position des parents vis-à-vis de la sexualité de la fille, où la virginité, gage de chasteté, est considérée comme une « valeur » et une « règle sociale à conserver » [27]. Cet héritage culturel transgénérationnel serait à l'origine, dans notre étude, d´un âge avancé du premier rapport sexuel superficiel ou pénétrant (17,23 ans et 16,86 ans, respectivement). Les étudiants en médecine en Inde avaient un âge moyen au premier rapport sexuel de 17,5 ans [17] et ceux en Amérique du Nord avaient un âge similaire lors du premier rapport sexuel (aux alentours de 18 ans), et ce, quel que soit leur sexe et leur orientation sexuelle [22]. Dans la littérature, l´âge au premier rapport sexuel peut varier de manière importante selon les pays [28] et tend à baisser durant les dernières décennies, comme le montrent des études consécutives menées dans différents pays [29,30].

**Limites de l’étude:** notre étude présente certaines limites qui doivent être précisées. Notre échantillon était, non seulement de taille réduite, mais aussi hétérogène (des niveaux d'étude différents, une répartition inégale entre les deux sexes). Le choix des questions concernant les connaissances en matière de sexologie n´a évalué que certains aspects de la sexualité. Par ailleurs, le caractère binaire des réponses proposées (vrai ou faux) ne permettait pas de nuancer les réponses des participants.

## Conclusion

A la lumière de notre étude, certains points méritent d´être soulignés. Des lacunes dans les connaissances en matière de sexualité existent chez les étudiants en médecine, du moins dans certains de ses aspects. Parmi les facteurs pouvant expliquer ce manque de connaissances, nous invoquons la carence d´une éducation sexuelle depuis le jeune âge, l´absence de programme d´information bien codifié en matière de sexualité et la ténacité des traditions et des tabous de notre civilisation, encore conservatrice quant à ce thème. Un programme complet et uniforme sur la sexualité humaine, ainsi que ses troubles, au niveau de la faculté de médecine de Sfax, peut sensiblement améliorer la capacité des futurs médecins de fournir des soins optimaux à leurs patients, chaque fois qu'ils sont interpelés pour des problèmes sexuels, et contribuer, ainsi, à la promotion de la santé sexuelle de notre société.

### Etat des connaissances actuelles sur le sujet

- Les sources d´information sur la sexualité sont très diverse;
- En dehors d´un enseignement postuniversitaire, les connaissances basiques en sexologie sont souvent éparpillées entre plusieurs matières lors des études médicales;
- Les attitudes des étudiants en médecine vis-à-vis de la sexualité et leurs pratiques sexuelles dépendent de leur contexte socioculturel.

### Contribution de notre étude à la connaissance

- Les principales sources d´information sur la sexualité chez les étudiants en médecine sont loin d´être des sources scientifiques;
- Des lacunes existent dans les connaissances en matière de sexualité chez les étudiants en médecine et sont, surtout, en rapport avec un héritage socioculturel;
- Un programme d´enseignement des bases fondamentales en sexologie doit être mis en place dans chaque faculté de médecine pour assurer une formation adéquate des futurs médecins.

## Conflits d’intérêts

Les auteurs ne déclarent aucun conflit d'intérêts.
